# Characterization of W–Cr Metal Matrix Composite Coatings Reinforced with WC Particles Produced on Low-Carbon Steel Using Laser Processing of Precoat

**DOI:** 10.3390/ma13225272

**Published:** 2020-11-21

**Authors:** Dariusz Bartkowski, Aneta Bartkowska, Paweł Popielarski, Jakub Hajkowski, Adam Piasecki

**Affiliations:** 1 Institute of Materials Technology, Faculty of Mechanical Engineering, Poznan University of Technology, ul. Piotrowo 3, 61-138 Poznan, Poland; pawel.popielarski@put.poznan.pl (P.P.); jakub.hajkowski@put.poznan.pl (J.H.); 2 Institute of Materials Science and Engineering, Faculty of Materials Engineering and Technical Physics, Poznan University of Technology, ul. Jana Pawła II 24, 60-965 Poznan, Poland; aneta.bartkowska@put.poznan.pl (A.B.); adam.piasecki@put.poznan.pl (A.P.)

**Keywords:** W–Cr coating, tungsten carbide, laser processing, microstructure, microhardness, X-ray diffraction (XRD), energy-dispersive spectrometry (EDS), corrosion resistance, wear resistance, nanoindentation technique

## Abstract

The paper presents the study results of laser processing of precoat applied on C30 steel. The precoat consisted of powder mixtures with a binder in the form of water glass. Tungsten powder, chromium, and tungsten carbide (WC) were used to produce the precoat. The laser processing was carried out using a Yb:YAG disc laser with a rated power of 1 kW. Constant producing parameters (power of laser beam, 600 W; laser beam scanning rate, 400 mm/min) were applied. Chemical composition of the precoat was a variable parameter in coating production. A mixture consisting of 50% W and 50% Cr as a metal matrix was prepared. Subsequently, WC particles in weight ratios of 25%, 50%, and 75% were added to matrix. As a result, W–Cr metal matrix composite coatings reinforced with WC particles were formed. This study focused on investigation of microstructure, microhardness, phase, and chemical composition as well as corrosion and wear resistance, of the newly formed W–Cr/WC coatings. An instrumented nanoindentation test was also used in this study. As a result of laser beam action, the newly formed coatings had an interesting microstructure and good properties which were improved in comparison to substrate material. It is anticipated that the resulting coatings, depending on the treatment parameters (e.g., W–Cr/WC powder mixture) used, can be successfully applied to metal forming or foundry tools.

## 1. Introduction

In order to characterize the properties of tool materials, the material should be analyzed in terms of its microstructure and a number of physicomechanical properties. When examining the microstructure, special attention should be paid not only to the material core, which must be characterized by both high hardness and high ductility, but also to the condition and properties of the material’s surface layer. Metalworking tools, i.e., tools intended for use in such technologies as machining, founding, or, above all, plastic working, are exposed to intense wear due to friction and very often to dynamic loads, which may cause their cracking. Therefore, the surface layer of the material should be refined with appropriate chemical elements to reduce tool wear. By extending the life of the tools, both the cost of their regeneration and the time needed for their replacement are reduced. Disassembly and assembly of damaged tools and tools for plastic working or foundry generate undesirable downtime and, thus, increase production costs. Improving the operational properties of tools is often associated with an increase in the microhardness, as well as wear resistance, of the surface layer. The improvement of operational properties is possible mainly thanks to methods and techniques of surface engineering [1,2]. It is economically justified as we do not replace the entire tool with one made of more expensive material, but only modify its top layer, which is damaged most quickly. Surface treatment processes lead to the creation of new and often unique properties, and this may positively affect the parameters of technological process.

In the literature, there are descriptions of methods and techniques of increasing wear resistance by friction of tools mainly through the use of ceramic materials [3,4,5,6,7,8,9,10,11,12,13,14,15,16,17,18], as well as diffusion processes, which may result in the formation of hard and wear-resistant carbides [19] or borides [20]. High-energy methods such as thermal spraying [21] or hardfacing [17,18] are also often used. It is on the methods using high-energy sources that the greatest hopes are currently placed [11,16,22,23,24,25]. Among these methods, the most important are plasma spraying [21] and laser cladding [3,4,5,6,7,8,9,10,11,12,13,14,15,16,25,26,27,28,29,30,31,32,33,34,35,36,37,38,39,40].

In [25], the authors produced an Ni-based composite coating reinforced with tungsten carbide (WC) particles. The authors formed this coating on a mild steel substrate using a high-power diode laser. They analyzed single-layer coatings contained different quantities of Ni60 matrix powder and W + C reinforcing powder. Additionally, a five-layer coating with different amounts of these materials was produced. The authors found that the multilayer coating was characterized by the highest microhardness among all coatings. The maximum microhardness value was about 3.7-fold greater than seen in the steel substrate material. The obtained coating was also characterized by the absence of pores and cracks. The author of [3] compared wear resistance between conventional hard facing and laser cladding in the coatings of nickel–chromium alloy reinforced with WC particles. The author stated that rapid solidification rate and low dilution obtained by the laser cladding method contributed to better resistance to abrasion. In this study, it was observed that, for metal matrix composite coatings reinforced with WC particles, the laser cladding method contributed to obtaining a finer microstructure and higher WC particle volume content. These features have a significant influence on microhardness and wear resistance. The authors of [27] prepared Fe-based amorphous composite coatings reinforced with a WC phase using laser processing. They stated that additional laser remelting can reduce porosities and cracks of the produced coating, as well as improve corrosion resistance. They noticed that the microhardness of the remelted coating was approximately 1.13-fold higher than without an additional remelting process. Furthermore, in [4], WC–Ni composite coatings obtained using laser processing were described. In this case, Ti-6Al-4V alloy was used as the substrate material. In this study, high-frequency microvibration was used to assist laser cladding of Ni-based composite coatings. The results showed that microvibration characterized by high frequency promoted uniform distribution of WC carbide in the (Ti)Ni eutectic solid solution. The authors observed that an appropriate increase in vibration frequency caused refinement of the grains, resulting in an increase in wear resistance of the composite coating.

The addition of WC particles is intended primarily to increase the hardness of the coating and its wear resistance. Many publications focused on laser cladding [3,4,5,6,7,8,9,10,11,12,13,14,15,16,25,26,27,28,29,30,31,32,33,34,35,36,37,38,39,40] or laser alloying [41] using hard materials for different applications where high microhardness or wear resistance are required, for example, agriculture [16,17,18]. The authors of [6] presented the microstructure, microhardness, and wear properties of Fe-based coatings reinforced with various contents of WC particles produced on H13 hot-working die steel using laser cladding technology. The authors found that a small number of the WC particles in the WC-added coatings melted, whereas the unmelted WC particles in the coatings played the role of hard reinforcement. At the same time, the nucleation effect contributed to refinement of coarse grains around WC particles. The increase in WC content in Fe-based coatings led to an improvement of wear resistance and microhardness. In [5], the authors produced the Fe–Cr–W–C surface alloy via application of an in situ reaction during the laser cladding process using WC and AISI 410 stainless-steel precursor powders. The authors observed that the particles no greater than 12 μm tended to overshoot their boiling temperature and, thus, not make it to the substrate, whereas the particles exceeding 30 μm tended not to achieve their melting point temperature. Such particles survived in the melt pool in solid form. The authors found that the carbide particles were dissolved relatively easily due to the free energy in the Cr–C–W system and formed Cr–C compounds.

In [28], the microstructure of Stellite-6/WC coatings obtained via the laser cladding method using a CO_2_ laser beam on steel was investigated. Powder mixtures with WC contents of 0%, 9%, 18%, 27%, 36%, 45%, 54%, 72%, and 100% were applied via dual powder feeding of Stellite-6 and WC powders using different feeding rates. The authors found two kinds of solidification in the obtained microstructure. The first was characterized by dendrites, as well as interdendritic eutectics (obtained with WC amounts ranging from 0% to 36%). In this case, the WC particles added to the coating were fully melted into the melt pool. The second was characterized by diversely faceted dendrites in flower, block, star, and butterfly shapes. This solidification was obtained for 45% to 100% WC content. It should be mentioned that most of the WC particles were melted, and the microstructure consisted of a resolidified WC phase and Co, as well as various complex carbides (Co–W–C/Fe–W–C). In [6,28,40], the mechanism influencing the microstructure of WC particles was also featured.

Recently, there has been increased interest in the use of laser processing methods to produce composite surface layers on steels. Such layers have a composite structure, i.e., they are composed of a metal matrix reinforcing with hard intermetallic phases. The most commonly used matrices involve iron [6,7,17,18,26], nickel [13,14,16,25,26,28,36,38,40], cobalt [33], and alloys containing these elements. These alloys are characterized by good wettability of the reinforcing phase, facilitating adherence of this phase. The most frequently used reinforcing particles are various types of high-melting metal carbides, e.g., tungsten carbide (WC) [3,4,5,6,7,8,9,10,11,12,16,21,26,27,28,30,36,37,40], silicon carbide (SiC) [13,14], boron carbide (B_4_C) [38,39], or titanium carbide (TiC) [42].

In this work, a study of composite coatings with a matrix of tungsten and chromium (W–Cr) reinforced with hard particles of tungsten carbide (WC) was carried out. The coatings were created on nonalloy carbon steel. The aim of the research was to obtain a layer with increased hardness and wear resistance so that it could be used as a coating to increase tool durability for plastic working and casting.

## 2. Materials and Methods

The W–Cr metal matrix composite coatings reinforced with WC particles were produced on C30 steel substrate. The chemical composition of this material is presented in Table 1. Cuboid specimens cut from a square bar with dimensions of 12 mm × 12 mm × 5 mm were used. The specimens were machined (milled and ground) to obtain a surface roughness corresponding to Ra = 1.25.

**Figure 1:**
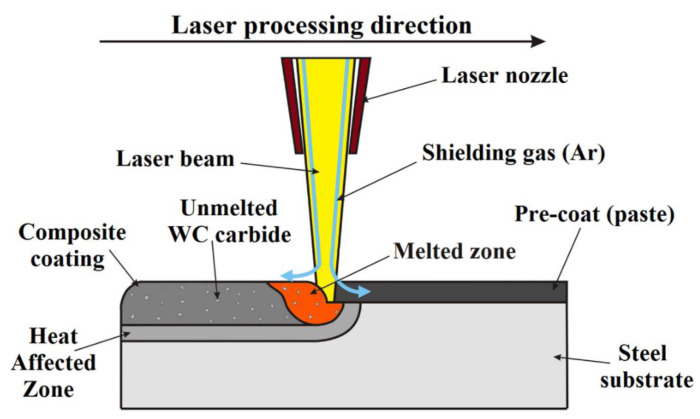
Scheme of laser processing.

To produce the coatings described in this work, several types of precoats in the form of paste with a thickness of 100 μm were used. The precoats were applied to the steel surfaces of specimens using a handheld applicator. This applicator was made from two hardened steel plates. The first plate had a slit with a height corresponding to the thickness of the precoat. The second plate (base plate) was equal to the height of the specimen. The specimen was placed in the hole of the base plate. Afterward, the paste was applied to the surface of specimen, and then the paste thickness was evened out using an applicator. The thickness was measured using an ultrasonic sensor. Pastes consisted of powder particles and a binder in the form of water glass. The powder mixture of tungsten and chromium was used as a matrix in a weight proportion of 50%/50%. In order to change the properties of W–Cr coating, 25%, 50%, and 75% WC particles were gradually added. A coating using only WC powder was also produced. The precoats were then processed using a Yb:YAG disc laser beam. The size of tungsten and chromium powder particles did not exceed 20 µm, and their shape was irregular, whereas the granularity of the reinforcing phase particles did not exceed 45 µm. Powder mixtures were prepared in a ball mill for 3 h, followed by drying at 100 °C for 1h. The specimens with prepared precoats were subjected to laser processing using a five-axis LaserCell 3008 laser device with a TruDisk Yb:YAG laser (TRUMPF, Ditzingen, Germany). The rated power of the laser used was equal to 1 kW. The laser beam was characterized by a circular cross-section and a diameter of 1.2 mm. The specimen was laser-processed on the entire surface with 75% track overlapping. The scheme of laser processing of the precoat applied on C30 steel, as well as the expected final metal matrix composite coating, is shown in Figure 1. The laser processing parameters are presented in Table 2. The aim of this study was to determine the effect of the chemical composition of the precoat on the properties of obtained coatings. The parameters were selected on the basis of preliminary tests.

**Figure 2:**
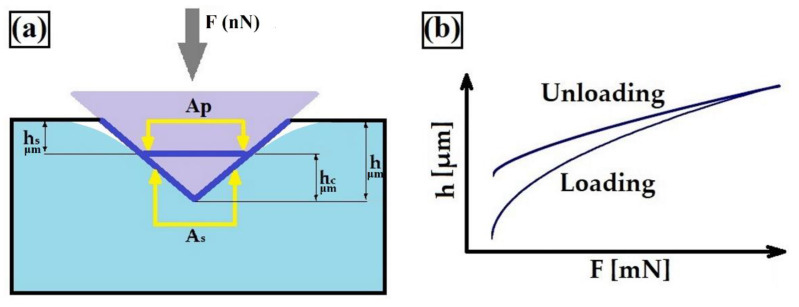
Methodology of the instrumented indentation test: (**a**) scheme measurement; (**b**) example of a typical load–displacement curve obtained during nanoindentation test.

Microstructure and macroscopic observations were carried out using two different scanning electron microscopes: VEGA 5135 (TESCAN, Brno, Czech Republic) and MIRA3 (TESCAN, Brno, Czech Republic). The first microscope was equipped with a Prism 2000 Si(Li) energy-dispersive X-ray spectrometer (Princeton Gamma Tech Instruments, Princeton, NJ, US) and PGT software Spirit 1.06 software. The other microscope was equipped with an EDS-UltimMax energy-dispersive spectrometer (Oxford Instruments, High Wycombe, UK) and Aztec Energy Live Standard software. The microstructure was determined on cross-sections, perpendicular to produced coatings. The specimens were first sanded on sandpaper with grit from 80 to 2500 and then polished using diamond paste. To reveal the microstructure of produced coatings, etching by “royal water” solution (HCl and HNO_3_ in a 3:1 ratio) was used. The phase analysis of W–Cr/WC coatings was performed on an EMPYREAN X-ray diffractometer (PANalytical, Malvern, UK). An angle range of 20° to 90° using Cu Kα radiation was applied. This kind of lamp has the ability to penetrate material up to 21 μm. A voltage of 45 kV and current of 40 mA were used. X-ray diffraction (XRD) analysis was performed at a temperature of 25 °C. Microhardness tests were carried out on cross-sections of coatings from the surface to the substrate. The microhardness tester FM-810 (Future-Tech, Kawasaki, Japan) equipped with an automatic indentation measuring system (FT-Zero software, Future-Tech, Kawasaki, Japan was used. All measurements were taken using an indentation load of 50 g. The loading time was 15 s. The electrochemical corrosion resistance tests were carried out using an ATLAS 1131 EU&IA device (Atlas-Sollich, Rębiechowo, Poland) in 5% NaCl aqueous solution. The potentiodynamic method was used. The corrosion potential and corrosion current of produced composite coatings were determined. These tests were performed at 25 °C with a scanning rate of 0.5 mV/s using a platinum electrode as an auxiliary electrode and saturated calomel electrode as a reference electrode. The results were determined on the basis of extrapolation of Tafel curves. Wear resistance was investigated using an Amsler-type device under dry friction conditions. A friction pair consisted of the plate-shape specimen with the produced composite coating and the ring-shape counter-specimen. The counter-specimen was made of 100Cr6 bearing steel after hardening. The hardness of the counter-specimen was 63 HRC. Wear resistance tests were performed using the following parameters: load, 400 N; rotation speed of counter-specimen, 179 rpm; time of friction, 210 min. The mass loss of specimens was measured using the AS220.R2 analytical balance (RADWAG, Radom, Poland) after every 40 min of wear measured with an accuracy of 0.0001 g. In order to determine the mechanical properties of coatings, a nanoindentation technique was used. This technique enables measuring mechanical properties such as the modulus of elasticity and hardness (Figure 2). A Fischer Picodentor HM 500 nanoindenter (Helmut Fischer, Sindelfingen, Germany) was employed in this study. The measurement stand was equipped with a vibration isolation plate, which dampened vibration occurring during work. In addition, the measuring device was enclosed in a thermoacoustic sheath, which reduced the effects of air flow and impact of acoustic waves. The test was performed in accordance with the ISO 14577-1 standard [43]. Measurements were performed with a 200 mN load applied for 5 s. In this study, a diamond indenter was used. The parameters from the instrumented indentation test were based on readings of the following data: indentation depth (h), contact depth (hc), elastic displacement (hs), projected area (Ap), and surface area (As), which are presented in Figure 2a.

## 3. Results and Discussion

### 3.1. Microstructure, Chemical, and Phase Analysis

**Figure 3:**
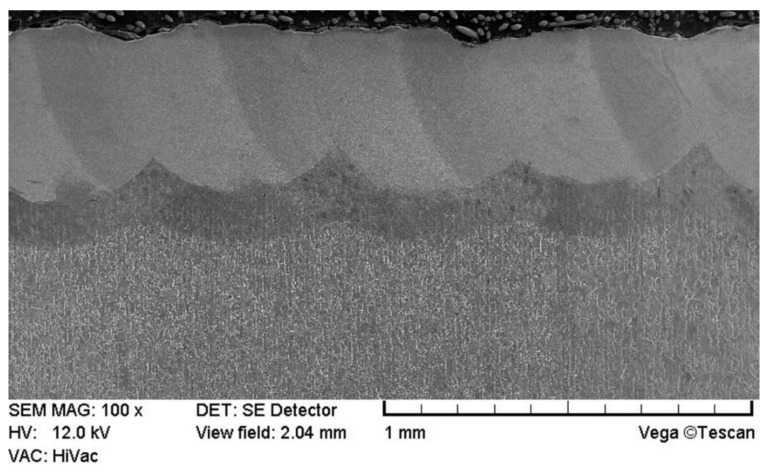
Microstructure of W–Cr coating produced using laser processing of precoat applied on steel substrate.

**Figure 4:**
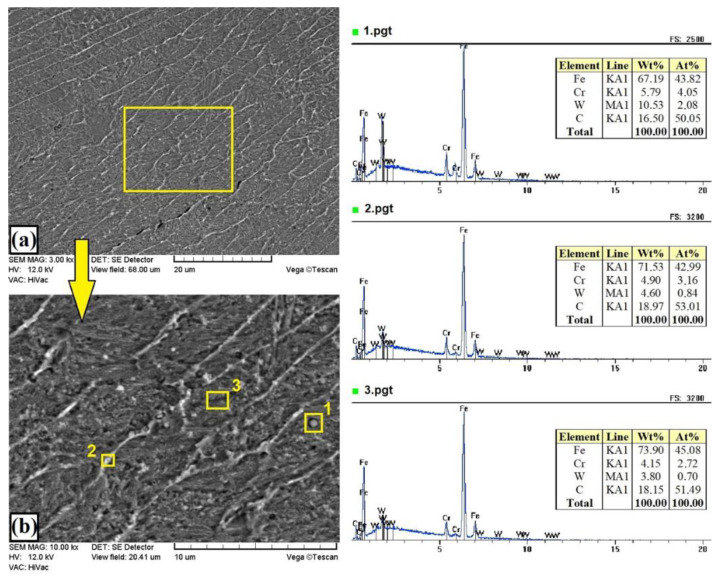
Microstructure and the energy-dispersive spectrometry (EDS) point analysis of W–Cr coating produced using laser processing of precoat applied on steel substrate: (**a**) melted zone; (**b**) magnification of melted zone.

The microstructures of the W–Cr coatings without reinforcing phase produced using laser processing of the precoat are shown in Figure 3. The microstructure of W–Cr coatings consisting of a melted zone obtained by the remelting of steel substrate and precoat could be identified. Below, the heat-affected zone (HAZ) was found. The remaining area shown in Figure 3 was ferrite–pearlite steel substrate. The microstructure of the melted zone consisted of the solid solution of chromium and tungsten in iron (Figure 3 and Figure 4). The W–Cr coating without a reinforcing phase had an average thickness of 430 µm including the heat-affected zone of approximately 170 µm. The coatings were more than four times thicker than the pre-coat, highlighting their high proportion of steel substrate. Produced coatings were characterized by good bonding with the steel substrate. The laser processing parameters used contributed to formation of a parabolic bond line between the coating and the substrate, which is characteristic for this kind of treatment.

Figure 4b shows the areas of chemical composition testing. The melting of the precoat caused the formation of carbide precipitates (square marked No. 1). In the remaining areas (dark and light), iron predominated, confirming its high content in the coating.

**Figure 5:**
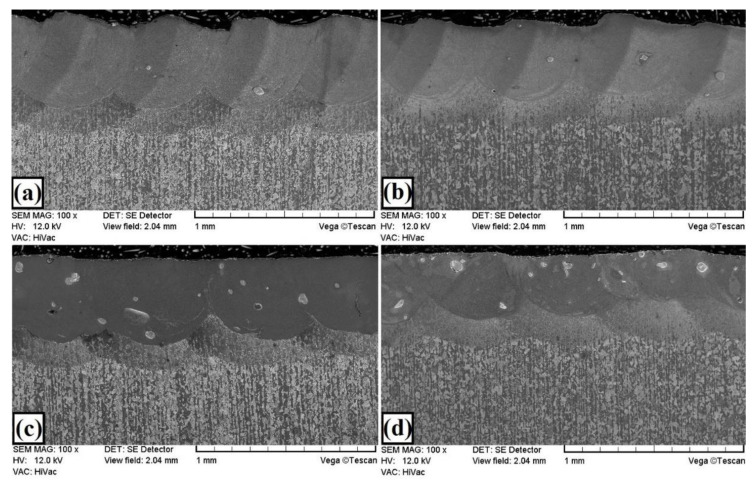
Microstructure of coatings produced using laser processing of precoat applied on steel substrate: (**a**) W–Cr/25% WC; (**b**) W–Cr/50% WC; (**c**) W–Cr/75% WC; (**d**) 100% WC.

The microstructures of the W–Cr/WC composite coatings with varying amounts of WC powder, as well as only using WC powder, are shown in Figure 5. As a result of precoat laser processing, composite coatings with a complex microstructure were obtained. The composition of the applied pre-coat had an influence on microstructure of coatings obtained. The microstructure consisted of the W–Cr matrix and unmelted primary WC particles. The proportion of these particles depended on their amounts in the precoat. When the proportion of WC reinforcing phase was equal to 25% and 50%, a very small amount of nonmelted WC particles in coatings was observed. This proves that the laser beam melted almost all components of the initial coating when the amount of WC phase was smaller. Increasing the content of reinforcing phase prevented larger WC particles from melting. The provided thermal energy was not sufficient to melt all the components of the precoat, which resulted in the formation of a W–Cr/WC or WC composite coating. Laser beam parameters were constant; however, due to the change in the chemical composition of the precoat, different geometries of the coating cross-sections were observed. Dendritic microsegregation was a common feature of all produced composite coatings. This was the effect of varying solidification rates of the melted zone over the thickness of coatings from the surface to the steel substrate. The changes in cooling rate were most visible in the case of specimens with the highest amount of WC reinforcing phase (Figure 5c,d). The WC particles became a kind of chill (as in a foundry) and accelerated the solidification process around their occurrence. The heat absorption by the high-melting carbides resulted in the production of thinner coatings. When there was a lower amount of WC particles in the precoat, heat was transferred to the steel substrate, and this contributed to the melting of substrate. This resulted in a higher proportion of iron in the coating, which in turn affected the properties of the produced coatings.

**Figure 6:**
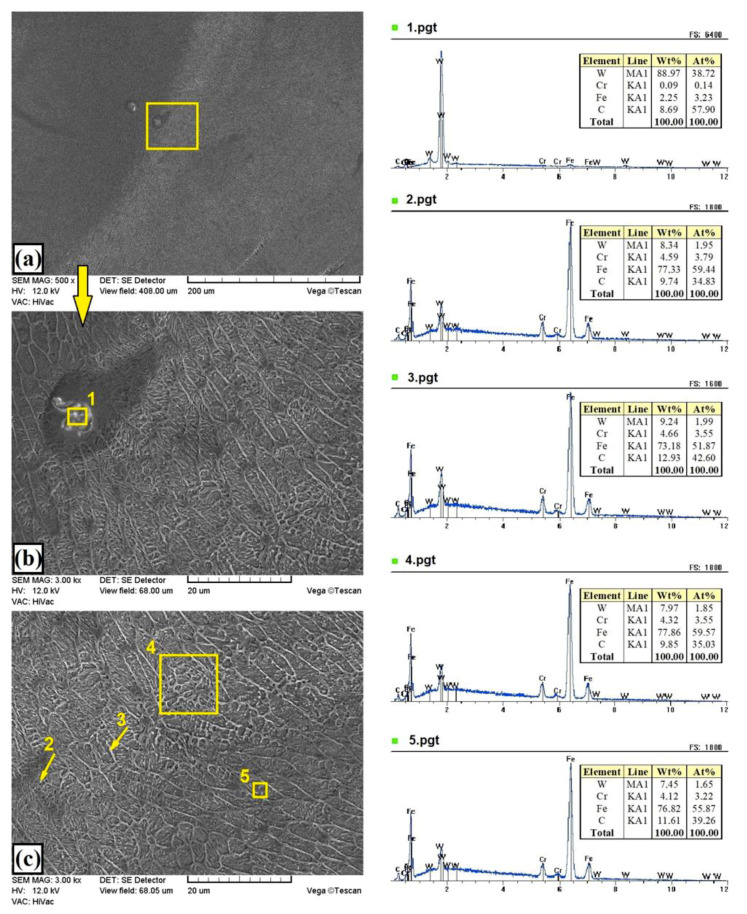
Microstructure and EDS point analysis of W–Cr coating reinforced with 25% WC particles produced using laser processing of the precoat applied on steel substrate: (**a**) general view of overlapping zone; (**b**) magnification of overlapping zone with unmelted WC particles; (**c**) matrix.

The addition of 25% tungsten carbide particles to the precoat contributed to an increase in thickness of the melted zone in the obtained coating to about 510 µm, while keeping the heat-affected zone at the same level, i.e., about 170 µm (Figure 5a). An unquestionable change in the microstructure of the obtained coatings was the presence of unmelted particles in the WC reinforcing phase. Thanks to this, coatings containing 25% of the WC phase could be called composite coatings. The particles were very rarely identified. It was found that only the largest particles did not melt (Figure 5a and Figure 6). The parameters of the laser beam used did not generate enough heat to melt the WC particles completely. Figure 6a–c show the melting zone along with the areas of chemical composition analysis, marked with squares. It is clearly visible that the unmelted particles were mainly composed of carbon and tungsten, corresponding to the primary WC carbides. On the other hand, the matrix was largely composed of iron with additions of tungsten and chromium. Thus, it can be stated that an Fe–W–Cr matrix was obtained. This proves the great influence of the steel substrate on the produced metal matrix composite coating.

**Figure 7:**
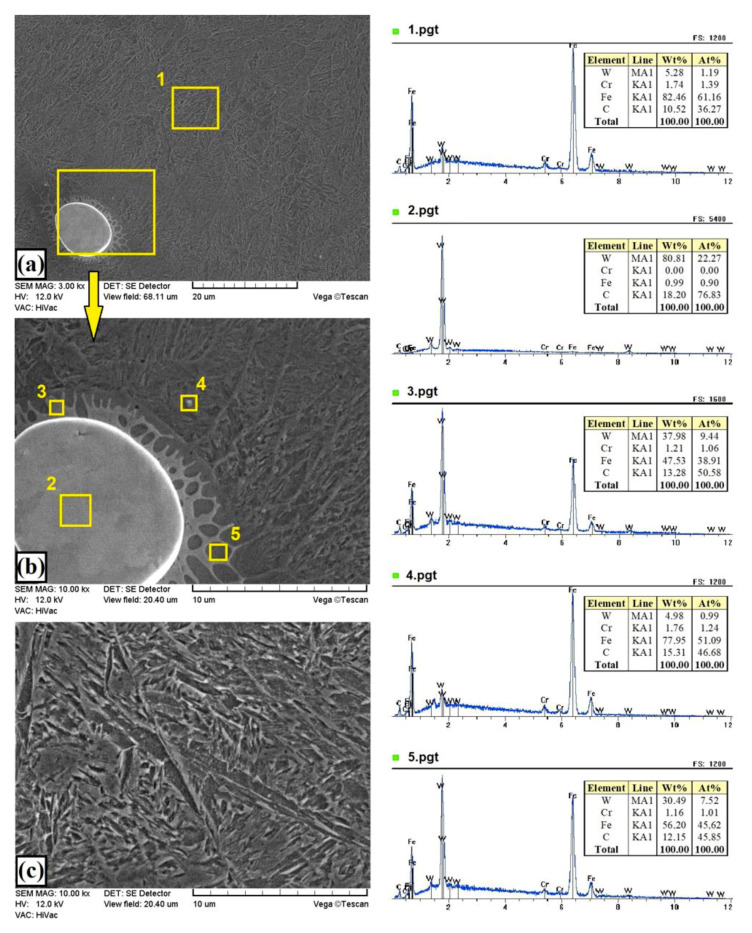
Microstructure and EDS point analysis of W–Cr coating reinforced with 50% WC particles produced using laser processing of precoat applied on steel substrate: (**a**) general view of the melting zone; (**b**) carbide-matrix interface; (**c**) matrix.

Figure 5b and Figure 7 show the microstructure of W–Cr /50%WC coatings. Increasing the content of the reinforcing particles caused a reduction in thickness of the produced coating by 10–20 µm. At the same time, increasing the content of the WC reinforcing phase resulted in an increase in the amount of unmelted primary WC particles. In the coating structure, no defects such as cracks or porosity were found, and the coating was very well bonded to the steel substrate. Figure 7a shows an example of a partially fused particle of the primary WC particle. The formation of new phases with a high content both of tungsten, iron, and carbon can be observed at the boundary of the carbide and the matrix (Figure 7b). These are probably complex secondary carbides of the M_7_C_3_ or M_2_C type. Between the dendrites of the newly formed secondary carbides, the analysis of the chemical composition indicated a high content of both iron (over 55 wt.%) and tungsten (over 30 wt.%). This is the darker area marked with square no. 5. The bright area around the partially melted primary WC carbide (marked with square no. 3) was characterized by increased amounts of tungsten and carbon. The microstructure of the remaining part of the melting zone was clearly characterized by a needle shape (Figure 7c).

**Figure 8:**
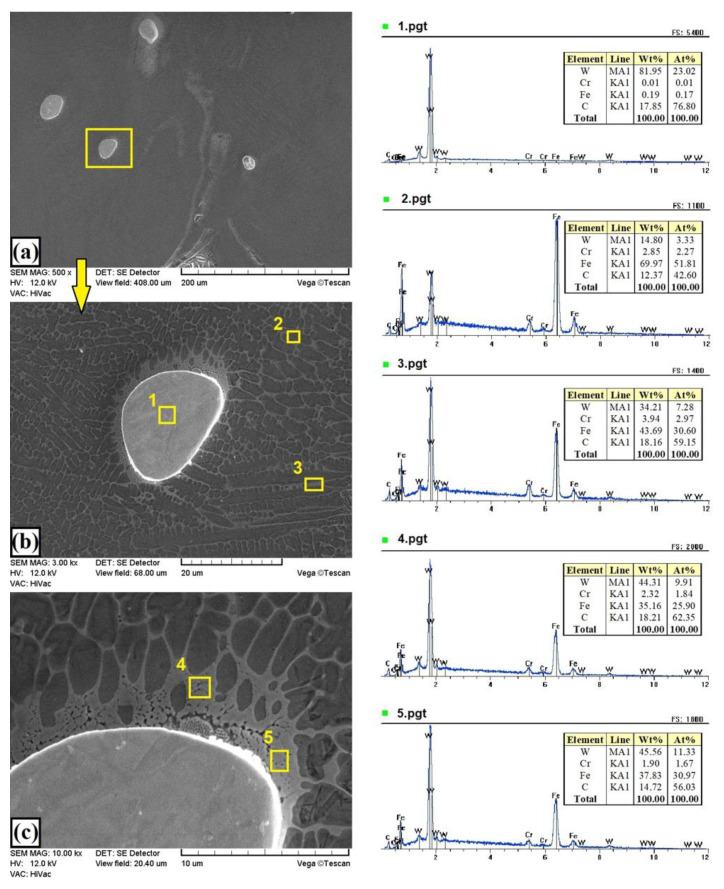
Microstructure and EDS point analysis of W–Cr coating reinforced with 75% WC particles produced using laser processing of precoat applied on steel substrate: (**a**) general view of the melting zone; (**b**) selected carbide; (**c**) carbide-matrix interface.

Figure 5c and Figure 8 show the microstructure of the W–Cr/75% WC coatings. Increasing the content of WC particles to 75% resulted in a reduction in the layer thickness to about 400 µm and a slight increase in the heat-affected zone to about 200 µm. The amount of unmelted primary WC particles increased (Figure 8a). Figure 8b,c show the boundary of WC carbide and the W–Cr matrix. A change in microstructure at the carbide–matrix interface was clearly visible. This microstructure had a significant impact on the properties of the produced coatings. In dark areas (e.g., marked with square no. 2), a much lower tungsten content was found than in bright areas (e.g., marked with square no. 3). In bright areas, there was a higher carbon content, which may mean that secondary carbides were formed there. The microstructure around large partially melted WC particles was more cellular, and many spherical precipitates of secondary carbides were formed. In each case, a small amount of chromium was found in the studied areas, but this may indicate the binding of chromium into complex carbides. A high content of tungsten and iron was also found in the dark areas of the matrix. This may be comparable to the Fe_7_W_6_ phase detected in XRD studies. It is clear from the observation of the microstructure that the increase in WC content in the case of W–Cr/WC coatings caused a reduction in the thickness of the produced coatings while maintaining a relatively constant thickness of the heat-affected zone.

**Figure 9:**
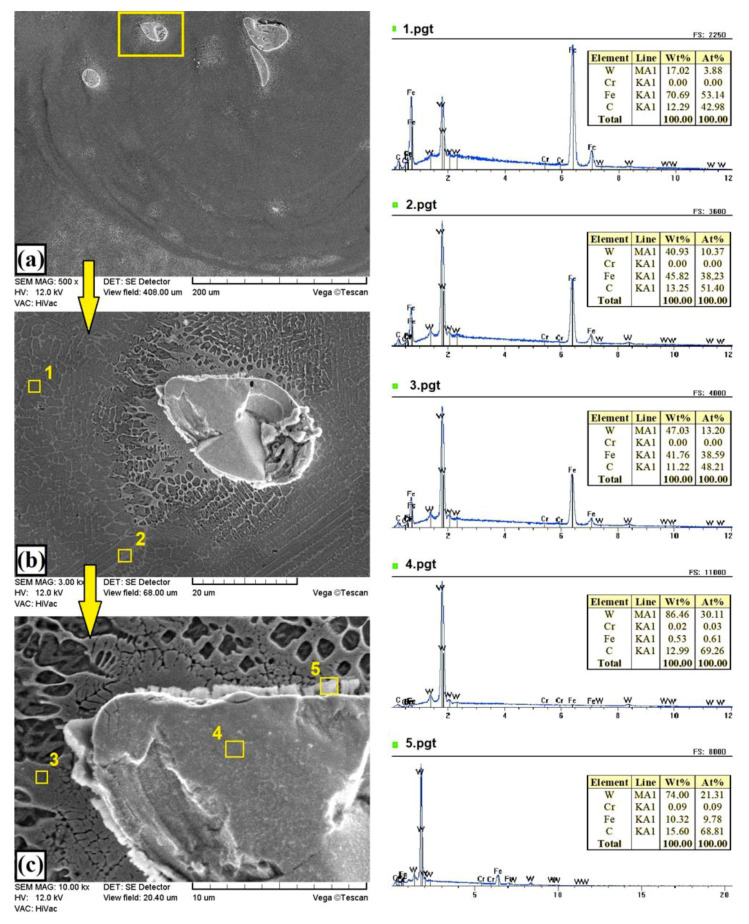
Microstructure and EDS point analysis of coating formed using 100% WC particles produced using laser processing of precoat applied on steel substrate: (**a**) general view of the melting zone; (**b**) selected carbide; (**c**) carbide-matrix interface.

The microstructure of the coating produced using only a WC precoat is shown in Figure 5d and Figure 9. In this coating, no porosity and no cracks were observed on the entire cross-section and entire surface. The thickness of the laser-modified surface layer was uniform. Larger-sized tungsten carbides did not melt completely, which resulted in a visible reinforcing WC phase, similar to that seen in the W–Cr/75%WC coating. However, the amount of large WC particles was higher in this case. As already mentioned, these coatings were characterized by the smallest thickness. This may be due to the very high temperatures necessary to melt the tungsten carbides. The heat delivered to the precoat was received by the tungsten carbide particles. Smaller WC particles melted, then mixed with iron, and finally formed a matrix, while larger WC particles received heat and only partially melted (only on the particle surface). The heat accumulated in the WC particles was then transferred to the steel substrate, which resulted in the formation of a fairly deep heat-affected zone, exceeding 220 µm in places. The coating, despite the absence of the W–Cr matrix, did not have cracks. It was characterized by a few porosities formed at the border of the tracks, i.e., in places where the material was most heated. Figure 9a shows an exemplary unmelted primary tungsten carbide. An enlargement of this area is shown in Figure 9b,c. Here, it is clearly visible that the surface of the primary WC carbide became the site of nucleation and growth of new phases (area marked with square no. 5). The chemical composition corresponded mainly with W_2_C and M_7_C_3_ phases.

In the case of the W–Cr coatings, very good cohesion between the coating and the substrate was found, which resulted from a good mixing of the coating material with the steel substrate. In the case of W–Cr/25%WC coatings, a high iron content was found, which also suggests a very good mixing of pre-coat with the substrate and very good metallurgical bonding. In the case of coatings containing 50% and 75% WC particles, a large amount of iron in the matrix and a very small amount of iron in the area of unmelted carbides were found. Coatings produced on the basis of pure WC precoat were characterized by the lowest iron content. This kind of coating was mainly composed of primary and secondary tungsten carbides or complex carbides containing iron.

**Figure 10:**
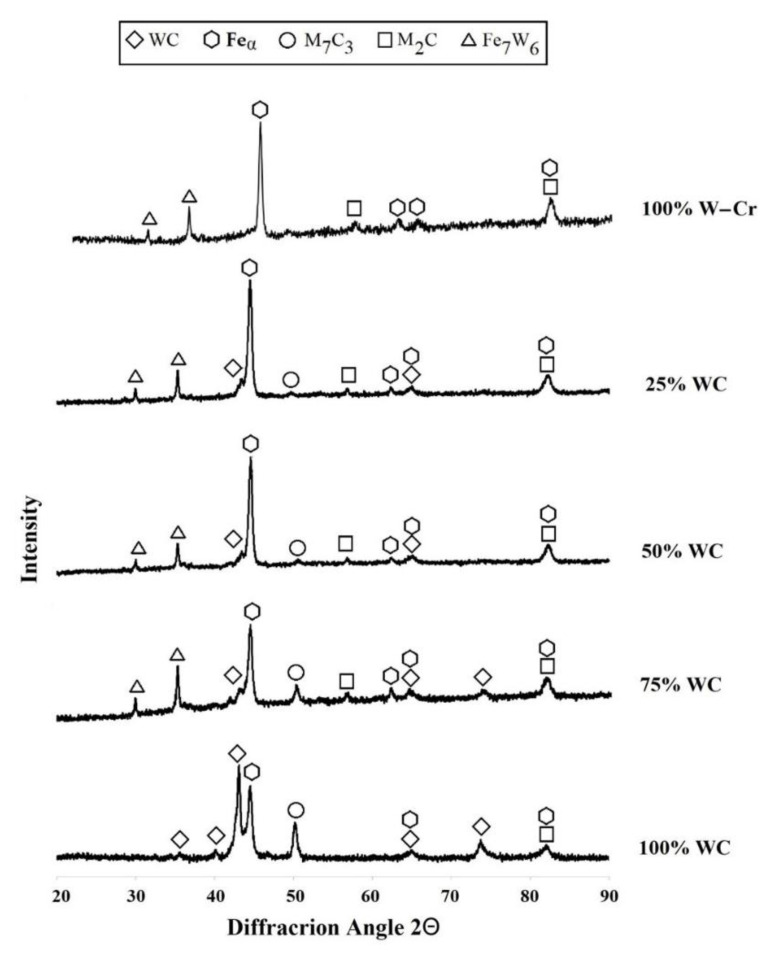
X-ray diffraction of coatings reinforced using laser processing of different precoat applied on steel substrate.

The phase composition of the produced W–Cr metal matrix composite coatings is shown in Figure 10. The presence of WC, W_2_C, M_7_C_3_, and Fe_7_W_6_ and Feα phases was found. The coatings produced via laser processing of the precoat containing a greater amount of the WC reinforced phase were characterized by a very intense peak of WC and M_7_C_3_ phases. These phases were only identified in coatings containing a WC reinforcing phase. The M_7_C_3_ phase was not identified in the W–Cr coatings. In all coatings, except for the WC coating, the presence of a Fe_7_W_6_ phase was found. It can be concluded that the intensity of this phase was comparable in all cases. Very intense iron peaks were found, which confirmed a high proportion of steel substrate in the coating. As WC particle content increased, the intensity of the different carbide phases increased. The results corresponded to the EDS microanalysis presented in Figure 4 and Figure 6, Figure 7, Figure 8 and Figure 9.

### 3.2. Microhardness Results

**Figure 11:**
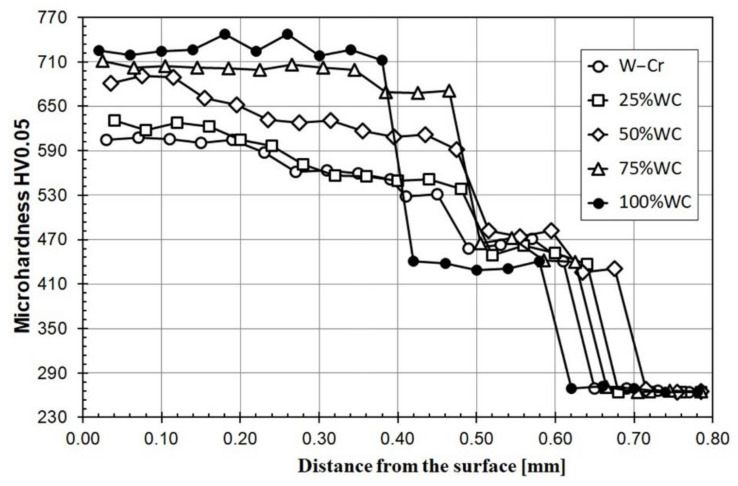
Microhardness of coatings produced using laser processing of different precoats applied on steel substrate (omitting the larger and unmelted WC particles).

The results of microhardness of the produced W–Cr metal matrix composite coatings reinforced with WC, as well as coatings produced using only W–Cr or WC precoat, are shown in Figure 11. Measurements of microhardness in the melted zone were performed by omitting the larger and unmelted WC particles. It was found that each coating had a melted zone whose microhardness was no lower than 500 HV0.05. In the case of W–Cr coatings without a reinforced WC phase, a very mild microhardness profile from the surface to the steel substrate was observed. The microhardness gradually decreased from about 600 HV0.05 to about 450 HV0.05 in the heat-affected zone. In the case of W–Cr metal matrix composite coatings reinforced with WC particles, the high microhardness of the melted zone (about 700 HV0.05) resulted from partial melting of WC particles. The addition of a hard reinforcing phase contributed to a microhardness increase in the entire coating. The addition of 25% WC (W–Cr/25%WC) did not significantly increase the microhardness in comparison to W–Cr coating. In these two cases, the microhardness profiles were very similar. A further increase in the content of WC reinforcing phase (to 50% and 75%) in the W–Cr matrix resulted in an increase in microhardness. However, if the unmelted WC particle microhardnesses were also taken into account, the average microhardness of these coatings would be much higher. It should be noted that the iron that came from the substrate also had an influence on microhardness. The steel substrate also melted and mixed with the precoat during the laser action. Therefore, the coatings produced in this way would have a lower hardness than carbide coatings produced by diffusion or, e.g., thermal spraying. This also applied to the coating made by laser processing of the 100% WC precoat. However, the microhardness profile of all produced coatings was very favorable due to the observed gradual decrease in microhardness from the surface to the substrate and an even microhardness in the area of the produced coating.

### 3.3. Corrosion Resistance

**Figure 12:**
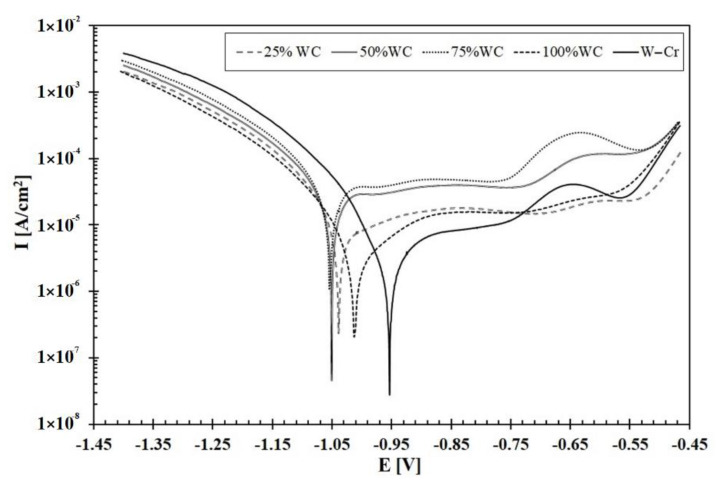
Corrosion resistance tests results of coatings produced using laser processing of different precoats applied on steel substrate.

The corrosion resistance test results of the W–Cr metal matrix composite coatings reinforced with WC particles, without WC particles, and with only WC are shown in Figure 12. The values of the electrochemical parameters (corrosion current and potential) were specified on the basis of Tafel curves extrapolation and are shown in Table 3. Analyzing the data from Table 3 and curves presented in Figure 12, it is visible that the smallest corrosion current was registered for the W–Cr coating. This indicated the high corrosion resistance of this coating. The WC coating was inferior only to the W–Cr coating taking into account the corrosion resistance. Much worse results in the corrosion resistance tests were recorded for the W–Cr metal matrix composite coatings reinforced with WC particles, as confirmed by the recorded greatest corrosion currents. After comparison of the corrosion resistance of all type of coatings, it was found that an increase in WC particle content contributed to a decrease in corrosion resistance.

**Figure 13:**
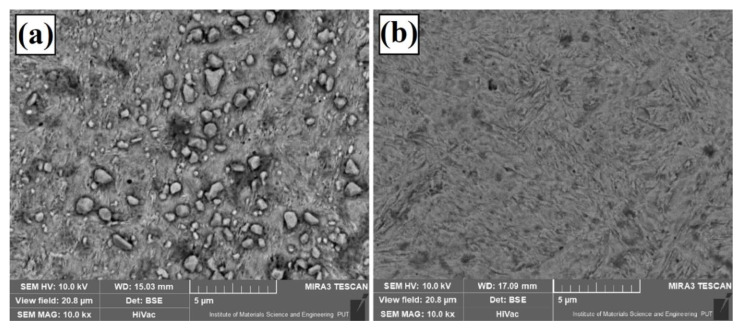
Image of surface condition after corrosion tests at large magnification (10,000×) for (**a**) W–Cr/75% WC coating and (**b**) W–Cr coating.

Figure 13 shows the alleged cause of the poor corrosion resistance of W–Cr/75% WC coatings. Observing the surface at very high magnification, a large number of secondary carbide precipitates with sizes not exceeding 1.5 μm could be identified (Figure 13a). In most cases, the size of the secondary precipitates was in the range of 0.3–0.6 µm. This created a very large number of corrosion cells contributing to the reduction in corrosion resistance. The dark areas on the surface were the places where corrosion was observed. These areas were much less common in the W–Cr coating (Figure 13b). It should be noted that the secondary carbide precipitates were not found in this coating.

**Figure 14:**
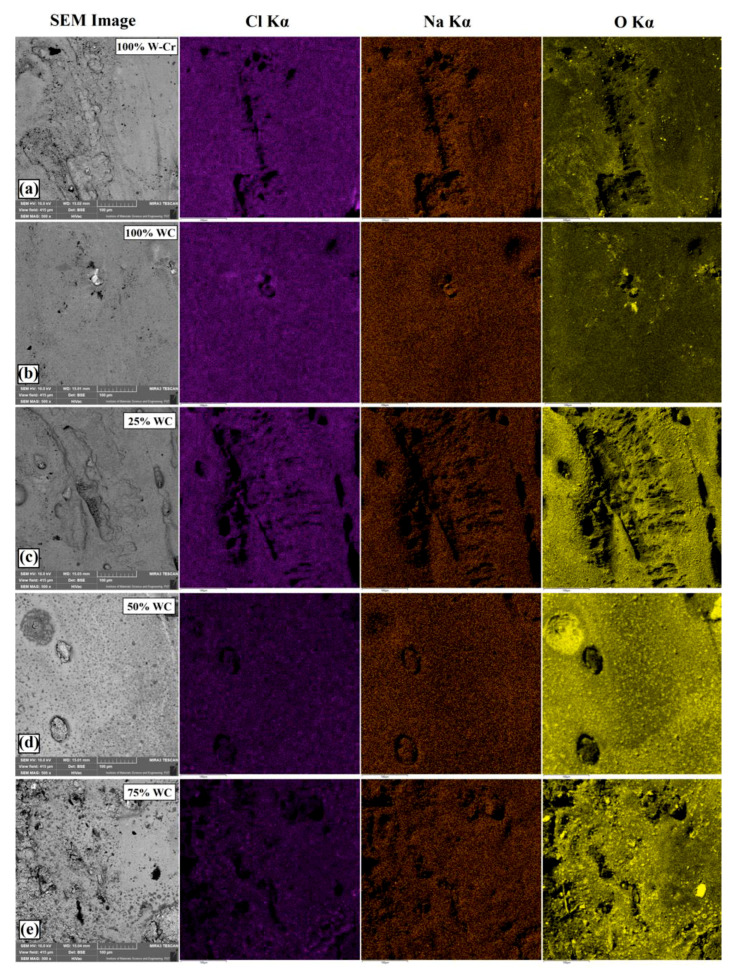
Macroscopic images of the surface condition after corrosion tests and EDS mapping for the following coatings: (**a**) W–Cr; (**b**) 100% WC; (**c**) W–Cr/25% WC; (**d**) W–Cr/50% WC; (**e**) W–Cr/75% WC.

The surface condition and results of EDS mapping for the specimens after corrosion resistance tests are shown in Figure 14. The most substantial effects of corrosion are shown in Figure 14c–e, where corrosion pits could be seen on large areas of the tested coating. In these cases, the entire surface of the coating was covered by oxides. Furthermore, an increased chlorine content was found, which was a component of the NaCl solution in which electrochemical tests were carried out. The EDS maps confirmed the obtained potentiodynamic test results. Therefore, it could be confirmed that the W–Cr coating was characterized by the highest corrosion resistance. Here, the amount of oxides formed on the surface was small. The addition of WC particles to the W–Cr matrix resulted in a reduction in corrosion resistance. In the case of the WC coating, good corrosion resistance was obtained, which was most likely due to the incorporation of corrosion-resistant tungsten carbides into the steel substrate without introducing other additional phases that could increase the possibility of producing corrosion cells.

### 3.4. Wear Resistance

**Figure 15:**
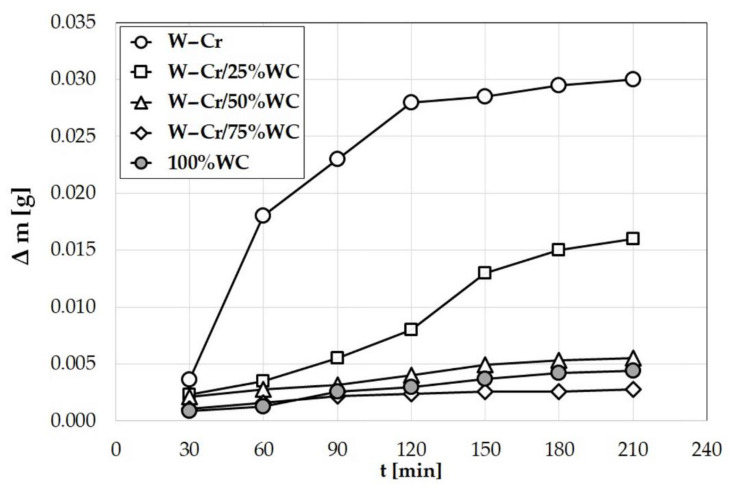
Wear resistance of produced W–Cr metal matrix composite coatings reinforced with WC particles.

Figure 15 shows the influence of the chemical composition of produced coatings on the wear resistance under dry friction conditions. It was found that the worst wear resistance was characteristic of coatings which did not have the reinforcing phase in the form of tungsten carbide particles. Even the addition of 25% WC particles contributed to a more than twofold decrease in the wear loss of the coating. Each successive increase in the amount of WC particles in the W–Cr metal matrix composite coatings had a positive influence on wear resistance. The W–Cr coating reinforced with 75% WC particles turned out to be the best among the specimens tested. The coating produced by laser processing of the WC precoat was slightly worse.

**Figure 16:**
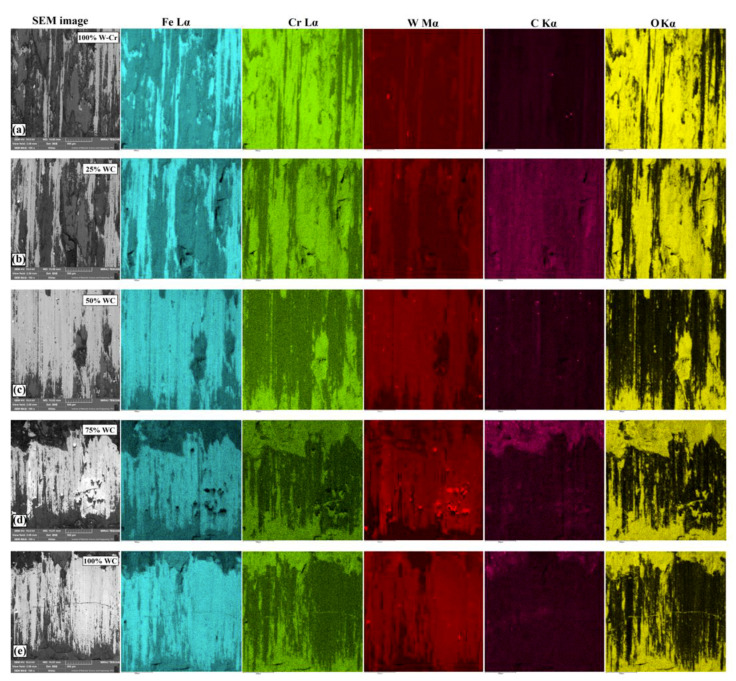
Macroscopic images of the surface condition after wear resistance tests and EDS mapping for the following coatings: (**a**) W–Cr; (**b**) W–Cr/25% WC; (**c**) W–Cr/50% WC; (**d**) W–Cr/75% WC; (**e**) 100% WC.

It was found that microcutting was the dominant destruction process in all analyzed coatings with WC reinforced particles. This phenomenon occurred when the coating was located in phases characterized by significant differences in hardness, e.g., hard microparticles or particles interacting like an abrasive. Microcutting after wear tests was parallel and was in accordance with the direction of the counter-specimen movement. In the case of analyzed samples with different percentages of WC, a proportional relationship between the microhardness and weight loss results could be observed. An increase in the coating microhardness contributed to obtaining smaller mass loss. Figure 16 shows the surface of the specimens after wear resistance tests, as well as EDS mapping results for produced coatings. For the W–Cr coating (Figure 16a), abrasive wear traces with grooving abrasion could be observed. In these areas, an increased content of oxides was found. Figure 16b–e show the surface condition of the specimens after wear tests and EDS mapping. These figures correspond to Fe–Cr/WC coatings. It can be seen that the coatings reinforced with WC particles were characterized by better wear resistance than the W–Cr coatings without a reinforcing phase. Significant signs of wear were observed in WC coatings. As a result of abrasion, significant stresses contributed to cracks. Cracks were not observed on the cross-section of coatings under the microscope; thus, they probably arose during wear tests. All specimens with composite coatings with varying WC contents were characterized by a lower content of oxidation products. The oxides formed on the surface were removed by microcutting with hard particles originating from the coating. The surface images after a wear test of WC–Ni laser-cladded coatings without or with vibration were presented in [4] where the authors found that the surface had numerous grooves. They found that the main type of coating damage was adhesive and abrasive wear. They also confirmed the formation of deep grooves in the surface after the wear test. However, no chemical changes were shown to be caused by abrasion. Moreover, in [6], the authors studied worn surfaces of Fe-based coatings reinforced with different contents of WC particles. The authors found that there were long, deep, and uniform grooves, indicating that microcutting was the main mechanism of wear. They found that, with increasing WC particle content, the main wear mechanism of the worn surface was abrasive wear. The resulting grooves were shallower and slenderer, and the adhesive wear degree gradually decreased.

### 3.5. Instrumented Indentation Test

**Figure 17:**
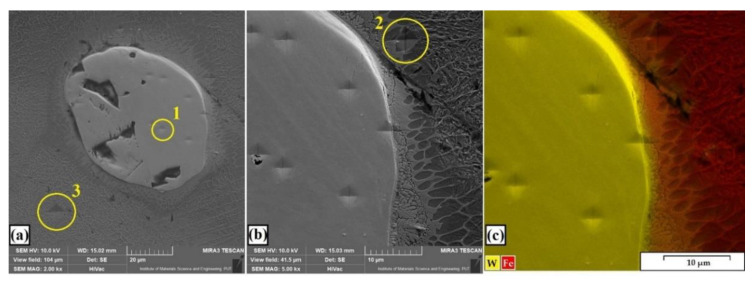
Example of microstructure within area of nanoindentation: (**a**) WC carbide in W–Cr matrix; (**b**) the boundary between carbide and matrix; (**c**) EDS mapping of nanoindentation area.

**Figure 18:**
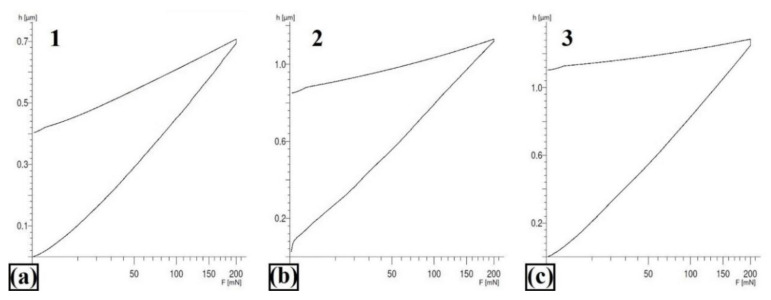
Example of the instrumented indentation test for W–Cr/50% WC coating; (**a**) in carbide; (**b**) boundary between carbide and matrix; (**c**) matrix.

Example areas of the nanoindentation test are shown in Figure 17, while Figure 18 shows an example of a load–indentation depth curve for the W–Cr/50% WC coating. The results of all measurements are presented in Table 4. The loading–unloading cycle was made for three indentations for all studied specimens, and all measurements were repeatable. It can be seen that the area around the carbide had increased hardness. It was related to the occurrence of secondary carbide phases growing at the carbide–matrix boundary. These phases had an increased tungsten content, as confirmed by the EDS mapping (Figure 17c). The produced coatings had a higher Young’s modulus than the steel substrate. This value was significantly increased by the introduced reinforcing phases, whose Young’s modulus was threefold greater than the value for steel. This study shows that the coatings were not very susceptible to plastic deformation, as reflected in the wear resistance. The W–Cr coating was characterized by the greatest plastic deformation. As the amount of WC particles in the W–Cr metal matrix composite coating increased, plastic deformation decreased. Plastic deformation reached the minimum values for coatings produced by laser processing of the precoat containing only WC particles.

## 4. Conclusions

Laser processing enables the production of composite coatings characterized by unique properties in a relatively short time in comparison to diffusion methods. According to the results of this study, the following conclusions can be drawn:

− increasing the content of WC particles reduces the thickness of the W–Cr/WC coatings;
− increasing the content of WC particles contributes to increasing the microhardness of the produced W–Cr metal matrix composite coatings;
− increasing the content of WC particles in the W–Cr metal matrix composite coatings contributes to increasing wear resistance;
− on the surface of the coating containing 100% WC, cracks were observed after a wear test. These cracks were probably formed during the friction process, because no cracks were observed during microstructure observation. These cracks led to reducing the wear resistance. Despite this, the wear resistance of 100% WC coatings was quite high;
− increasing the amount of WC as the reinforcing phase in the W–Cr composite coating reduced corrosion resistance. This was due to an increase in the number of corrosion cells in the entire coating;
− as a result of laser processing of precoats consisting of W, Cr, and WC particles, newly formed coatings rich in primary and secondary tungsten carbides (WC, W_2_C, M_7_C_3_) were obtained. These were carbide phases characterized by high microhardness.

## Figures and Tables

**Table 1:** Chemical composition of C30 steel used in study (wt.%).

C	Mn	Si	P	S	Cr	Ni	Mo	Fe
0.32	0.63	0.29	0.03	0.04	0.21	0.27	0.07	base

**Table 2:** Parameters of laser processing.

Composition of Powder Mixture		Parameters of Laser Beam	
W–Cr (%)	WC (%)	Power of Laser Beam (W)	Scanning Rate of Laser Beam (mm/min)
100	0	600	400
75	25	600	400
50	50	600	400
25	75	600	400
0	100	600	400

**Table 3:** Corrosion current and corrosion potential of composite coatings produced using laser processing of precoat.

Coating Type	Current I_corr_ (A·cm^2^)	Potential E_corr_ (V)
W–Cr	2.72 × 10^−6^	−0.953
W–Cr/25% WC	5.68 × 10^−6^	−1.04
W–Cr/50% WC	1.74 × 10^−5^	−1.05
W–Cr/75% WC	2.22 × 10^−5^	−1.05
WC	2.31 × 10^−6^	−1.01

**Table 4:** Average results of the instrumented indentation test for produced coatings for the following areas: carbide, boundary between carbide and matrix, and matrix.

Type of Coating	Designation	Martens Hardness (HM), N/mm^2^	Vickers Hardness HV	Young’s Modulus (E), GPa	Plastic Deformation η_plast_, %
100% W–Cr	Matrix	2154	256	186	85.5
W–Cr/25% WC	Carbide–boundary matrix	13,502 4889 4300	1967 643 520	556 242 276	62.9 76.3 84.4
W–Cr/50% WC	Carbide–boundary matrix	14,048 5506 4453	2164 748 525	527 247 316	59.1 72.3 85.5
W–Cr/75% WC	Carbide–boundary matrix	14,692 5775 4756	2259 807 589	582 232 285	59.6 70.2 79.6
100% WC	Carbide–boundary matrix	15,342 6349 6027	2385 930 857	602 225 229	60.4 63.1 67.9
